# GIS-based classroom management system to support COVID-19 social distance planning

**DOI:** 10.1007/s43762-022-00040-3

**Published:** 2022-05-28

**Authors:** Trupti Lokhande, Xining Yang, Yichun Xie, Katherine Cook, Jianyuan Liang, Shannon LaBelle, Cassidy Meyers

**Affiliations:** 1 Department of Geography and Geology, Eastern Michigan University, Ypsilanti, MI 48197 USA; 2 Institute of Geospatial Research and Education, Eastern Michigan University, Ypsilanti, MI 48197 USA

**Keywords:** Classroom management, COVID-19, GIS, Python, Seating capacity, Social distancing

## Abstract

Schools across the United States and around the world canceled in-person classes beginning in March 2020 to contain the spread of the COVID-19 virus, a public health emergency. Many empirical pieces of research have demonstrated that educational institutions aid students’ overall growth and studies have stressed the importance of prioritizing in-person learning to cultivate social values through education. Two years into the COVID-19 pandemic, policymakers and school administrators have been making plans to reopen schools. However, few scientific studies had been done to support planning classroom seating while complying with the social distancing policy. To ensure a safe return to campus, we designed a ‘community-safe’ method for classroom management that incorporates social distancing and computes seating capacity. In this paper, we present custom GIS tools developed for two types of classroom settings – classrooms with fixed seating and classrooms with movable seating. The fixed model tool is based on an optimized backtracking algorithm. Our flexible model tool can consider various classroom dimensions, fixtures, and a safe social distance. The tool is built on a python script that can be executed to calculate revised seating capacity to maintain a safe social distance for any defined space. We present a real-world implementation of the system at Eastern Michigan University, United States, where it was used to support campus reopening planning in 2020. Our proposed GIS-based technique could be applicable for seating planning in other indoor and outdoor settings.

**Supplementary Information:**

The online version contains supplementary material available at 10.1007/s43762-022-00040-3.

## Introduction

The coronavirus disease 2019 (abbreviated as COVID-19) outbreak has dramatically changed everyone’s life around the world. It disrupted every sector of the economy, affected public health, and made unforeseen and profound changes to social activities. The education system was no exception to it. As of January 2022, the United Nations Educational, Scientific, and Cultural Organization (UNESCO) Institute for Statistics has reported globally around fifty-four million students are affected by school closures caused by COVID-19. UNESCO stated this global disruption in education is the worst education crisis in history. While school closure was chosen as a priority strategy to reduce the transmission of COVID-19 as it reduces social contacts between students (Jackson et al. 2016), concerns were raised about the policy’s negative impact on society since the beginning of the pandemic. Researchers argued that school closure might cause economic harm to working parents, healthcare workers, and other essential workers being forced from work to childcare (Viner et al., 2020). Scholars also raised the concern that the extended out-of-school time due to the COVID-19 pandemic may exacerbate other health issues such as the increased risk of weight gain among children and a range of psychological harms due to social isolation (Brooks et al., 2020; Rundle et al., 2020). Moreover, being two years into the pandemic, a need to reopen schools and colleges has been advocated more than ever.

As the United States is slowly returning to in-person classes, policymakers and administrators in education systems need to consider how to reopen in a way that will keep students and staff safe. Among various guidelines issued by governmental agencies, a widely adopted one in the United States is the social (physical) distancing strategy from the Centers for Disease Control and Prevention (CDC). Following several studies, CDC has updated its recommendation on social distancing (CDC, 2021). It recommends maintaining a physical distance of at least 6 ft from other people who are not from the same household if an individual is not fully vaccinated. With additional preventive measures such as wearing face masks within classrooms, new CDC guidance encourages schools to maintain a physical distance of at least 3 ft between students.

There is now overwhelming evidence that the wrong design of indoor seating or failure to comply with the six-foot rule plays a dominant role in the spread of COVID-19 (Bazant & Bush, 2021; Miller et al., 2021; Shen et al., 2020). While educational institutions across the country are considering reopening for in-person learning, enforcing the social distancing strategy in the classroom has become crucial in the reopening plan. This also challenges school administrators in deciding how to adjust the classroom capacity and allocate the seating area within a limited classroom space to guarantee that each person is physically distanced from another as per CDC guidance. To the best knowledge of the authors, there is a lack of application in classroom management to support COVID-19 social distancing planning.

To fill this gap, we developed a geographic information system (GIS) based classroom management system to facilitate the decision-making process of school reopening. This paper focuses on the development of a GIS system for classroom management and planning to cope with the COVID-19 social distancing policy. We aim to demonstrate effective utilization of – GIS as an information system for modeling and managing campus classrooms; spatial analysis as an asset in evaluating the classroom capacity under various types of settings for social distancing; and GIS platforms as a means for communicating allocation of seats and rearrangements within classrooms with the help of maps.

This paper begins with the current status of COVID-19 pandemic. Section two will explore the impact of the COVID-19 pandemic on education, followed by a brief review of geospatial technologies/applications in COVID-19 and prior building information modeling. Section three will present materials and methodology. Results for the classroom GIS system will be illustrated and discussed in section four. Conclusions will be included in the final section.

## Literature review

### Impact of school closures

The outbreak of COVID-19 in early 2020, forced countries to take drastic measures like curfews, lockdowns and shelter-in-place to contain the spread of the virus. Non-essential workplaces were closed and moved to remote-work culture. Likewise, few education institutions switched to remote learning while others remained closed. Though, remote learning (online classes) became a tool for education during the pandemic, it did not show promising results. Remote and hybrid education requires access to digital technologies and internet connections. The United Nations International Children’s Emergency Fund (UNICEF) and the International Telecommunication Union (ITU) reported that two-thirds of world’s student population do not have internet connectivity at their home. This ‘digital divide’ in community is more acute in low-income regions such as sub-Saharan Africa (UNICEF-ITC, 2020). Several studies indicate a rise in dropout among public school students as a result of disrupted learning during pandemic in India. Moreover, this disruption also caused health impact by depriving many students of nutritious meals served at public schools (UNICEF and UNESCO, 2021). Considering the adverse impacts of school closures caused due to pandemic, United Nations has urged to make education a priority to achieve sustainable development goals of nations (UNGA, 2021).

The COVID-19 pandemic has also posed unique challenges for the U.S. educational system. School closures have impacted a total of 55 million students across the U.S. during March and April of 2020 (Kuhfeld et al., 2020). During this period, most public schools offered a virtual learning curriculum in place of traditional in-person learning. However, the switch to virtual learning was expected to have unintended consequences for students’ educational achievement. Studies showed that almost every student has faced difficulties with their mental health and well-being as a result of the pandemic (OCR, 2021). The U.S. administration plans to ensure safe reopening of schools by taking measures such as vaccination, indoor masking, regular screening/testing for COVID-19, and maintaining social distance. Meanwhile, schools from K-12 to the university level continue to grapple with classroom management questions for safe seating arrangements.

### Geospatial applications during pandemic

Geospatial technology has been employed in a variety of ways to assist in the mitigation, control, and understanding of the COVID-19 pandemic (Franch-Pardo et al., 2020). China’s National Health Commission created a mobile application to track activities and location to determine if the individual has come in contact with another individual who has tested positive for COVID-19 within the previous two weeks. Digital contact tracing could reduce the labor required by traditional contact tracing techniques (Kamel Boulos & Geraghty, 2020). Liu et al. (2020) studied a possible correlation between climatic features and the number of COVID-19 cases and geographic spread in Chinese cities. Various institutions have created interactive web-based maps to show the case counts of COVID-19 throughout the world, and at the state and county level in the United States. These interactive maps conveyed spatial disease trends and the extent to which COVID-19 has affected various communities (Dong et al., 2020). Smith and Mennis (2020) highlighted how GIS can be used to determine community at risk for higher infection and mortality rates based on the community’s environmental, health, and socioeconomic characteristics. Geospatial technology has also been used to report on population mobility and the effectiveness of stay-at-home orders by analyzing travel patterns via cell phone data (Gao et al., 2020; Huang et al., 2022) and social media data (Huang et al., 2020). In addition to digital contact tracing and determining communities at high-risk for COVID-19 outbreaks, Gibson and Rush (2020) used GIS to map and analyze informal settlements in Cape Town, South Africa to determine if social distancing is possible considering the close proximity of dwellings within the settlements. Orea and Álvarez (2022) used GIS to illustrate the effectiveness of lockdowns at the beginning of the COVID-19 outbreak in Spain, and simulated the outbreak’s trajectory depending on the lockdown date.

Previous publications have focused on the use of geospatial knowledge and GIS applications to assist with community and nationwide COVID-19 mitigation strategies and reporting. This paper addresses the lack of publications utilizing GIS for classroom management during the COVID-19 pandemic. The framework used to model classroom layouts and revised seating arrangements can be applied in any number of classroom or indoor settings as part of a mitigation strategy to maintain social distance between people and make gatherings of people as safe as possible.

## Data and methods

**Fig. 1:**
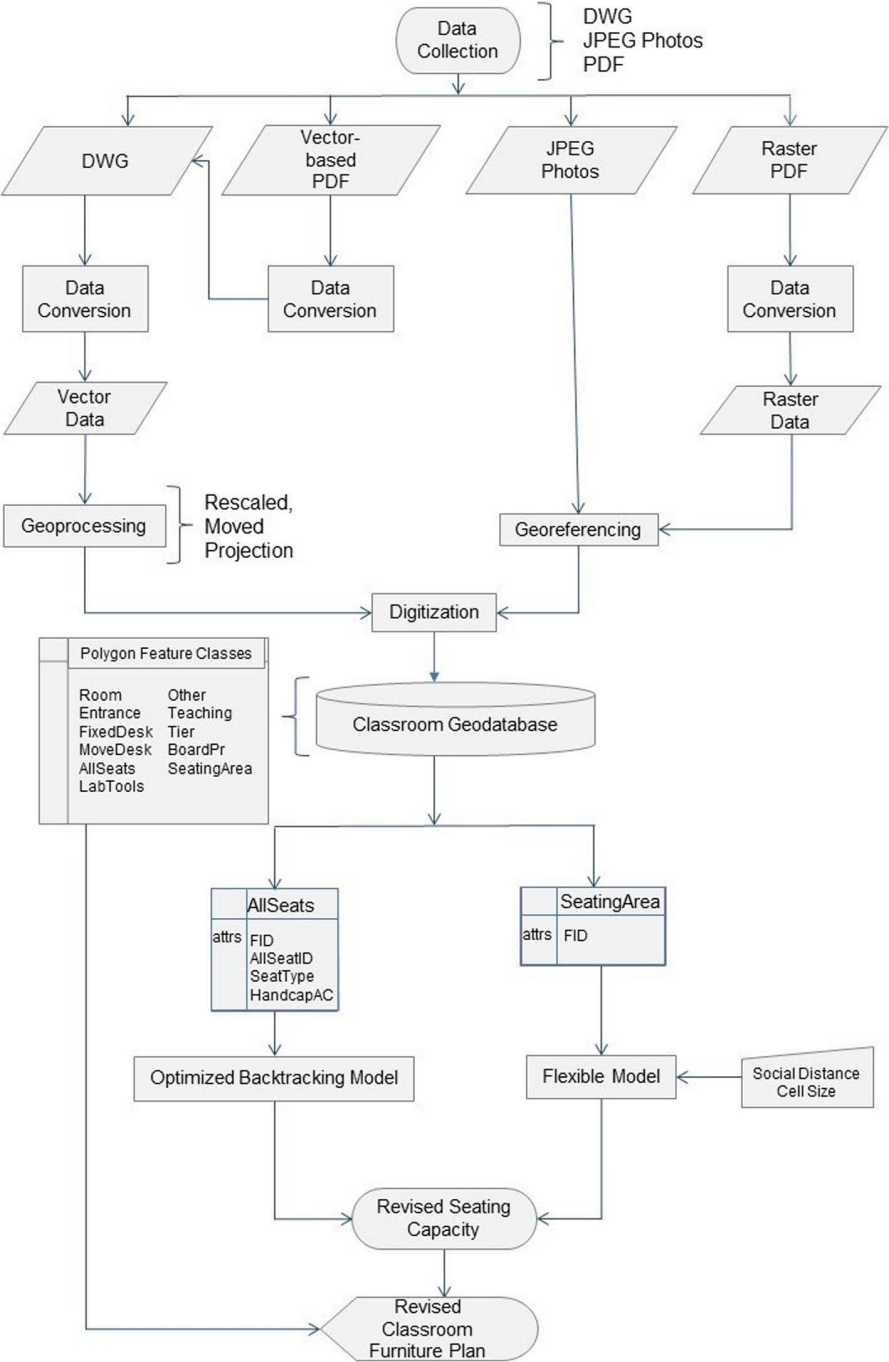
Study workflow for GIS-based classroom management system to support Covid-19 social distance planning

We used building plans for classrooms management at Eastern Michigan University (EMU) for our case study. The AutoCAD software was used to convert building plans (PDF) into readable files for ArcMap or ArcGIS Pro software. The study workflow shown in Fig. 1 can be described in two phases. Phase one consisted of data acquisition and processing, followed by tool development and analysis in the second phase. Phase one accomplished the preparation of classroom data from file formats such as JPEG, PDF, and DWG files. Each of the real-world features used in our analysis was a discrete object with a defined perimeter and area, making the polygon vector model an appropriate representation of these features within the GIS environment.

The polygon vector model allowed for the use of fill colors to generate easily recognized features as well as the ability to toggle features on and off for improved clarity and visualization. The classroom geodatabase served as the primary data for phase two. We developed two ArcPy based tools for spatial analysis in a classroom setting – fixed model backtracking tool and flexible model seating capacity tool. The fixed model backtracking tool was based on an optimized backtracking algorithm and produced an adjusted seating capacity for fixed-seating classrooms. While the flexible model seating capacity tool was a fishnet-based tool with user-specified input for social distancing parameters and calculated revised seating capacity for classrooms with movable furniture. The polygon features with the attributes required to run the tools are described in Table A1 of Appendix A. The culmination of this geospatial analysis was the production of revised seating capacity maps which allowed us to visualize the altered seating capacity for the analyzed classrooms. The details of the tool development will be discussed in the following section.

### Backtracking algorithm

The backtracking algorithm is one of the most significant algorithms for computational constraint satisfaction problems (Biere et al., 2009; Gurari, 1999),. From the perspective of searching, the backtracking algorithm enumerates a set of partial candidates step by step. All the possible solutions are tested, and a good solution is chosen as a selection. During the process, if the selection is found to be unsatisfied, it will be abandoned, and then, the process will track back to its previous step or even more steps and test the next available solution. Consequently, one of the two results will occur after operating the previous steps repeatedly: 1) One or more existing solutions to the whole set is found; 2) the question is unsolvable even though all possible steps have been tried (See Fig.A1 of Appendix A for pseudo code of optimized backtracking model). The eight queens puzzle is used as an illustration for the backtracking algorithm where space is defined by arrangements of eight chess queens on a standard chessboard of eight rows and columns.

### Fixed model backtracking tool

**Fig. 2:**
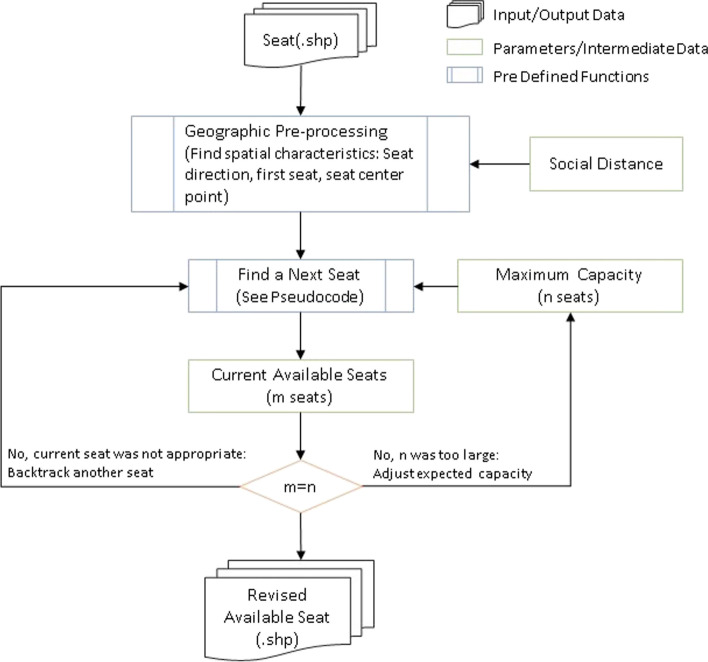
The flowchart of fixed model backtracking model

In this paper, we applied an optimized backtracking model (OBM) to solve the maximum seating capacity problem for the fixed classroom. In our fixed model backtracking tool, the space was the dimension of the fixed seats in the classroom, and the constraint rule was the social distance of 6 ft. ArcPy, a Python based package for performing ArcGIS function, was applied to build this model. Figure 2 provides a brief description of OBM workflow.

The tool would set a potential capacity at the beginning and would get adjusted during the process, until an actual maximum capacity was obtained. Additionally, the direction of the classroom, which would affect the starting location of the model, was participated as a parameter. Eventually, the maximum capacity and a revised available seating plan would be exported as the outcome of the model.

### Flexible model seating capacity tool

The flexible model seating capacity, a Python-based tool, was developed in ArcGIS software to estimate the classroom seating capacity and revise the classroom furniture plan based on specified social distancing parameters. With minimal user data inputs, the two prominent user-specified inputs required by the tool were – polygon shapefile representing seating area within a classroom, and distance required to be maintained between each seat. The tool would ensure that each seat in the revised classroom furniture plan will be at a specified social distance.

**Fig. 3:**
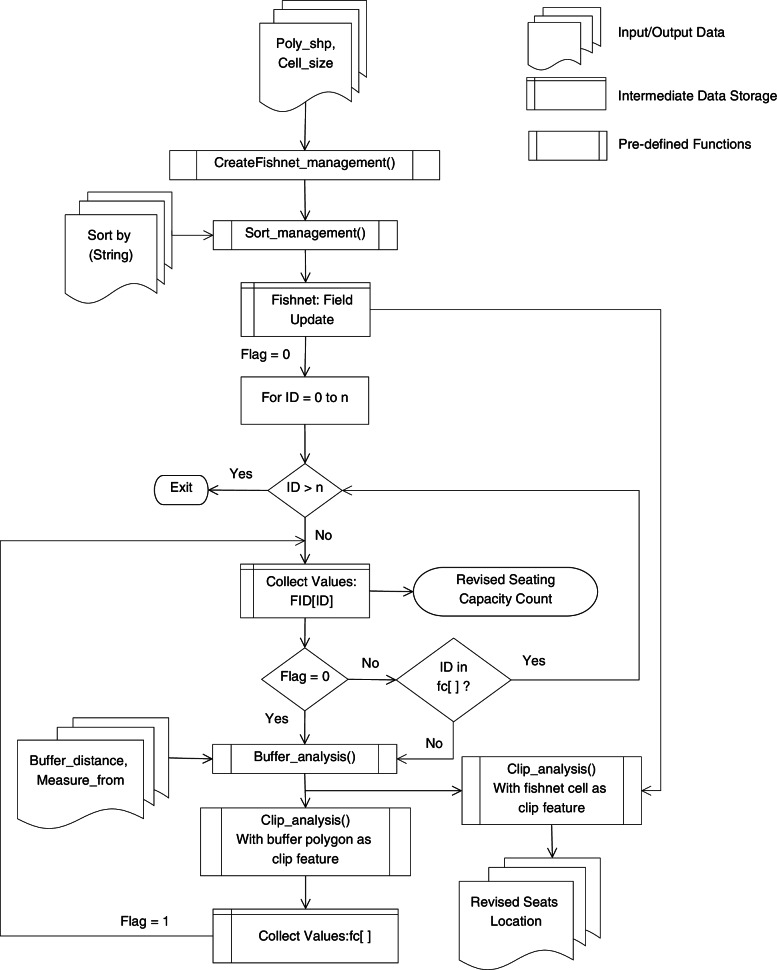
The flowchart of flexible model seating capacity tool

The tool mainly used three pre-defined ArcPy functions – CreateFishnet_mangement(), Buffer_analysis(), Clip_analysis(). The algorithm was realized by calling the ArcPy site package in Python script (ESRI, 2019). The workflow to automate geoprocessing the classroom data is shown in Fig. 3 (See Fig.A2 of Appendix A for pseudo code). The first process performed by the tool was to create a fishnet grid for a user specified polygon shapefile (Hyslop, 2012). The primary output of CreateFishnet_mangement() was a rectangular cell grid based on user specified cell size that represented the dimension of chairs in the classroom. The algorithm was designed to select a cell in the grid for its suitability concerning the COVID-19 social distancing measure. The tool provided flexibility in using different values and units of measurements for social distances to perform buffer analysis. The cells over which buffer analysis was performed got reserved as suitable or safe locations for seating. The output feature class obtained from this tool was a polygon shapefile representing revised furniture plan for the classroom.

## Results and discussion

We digitized 91 rooms across EMU’s campus and modeled revised seating capacity based on 6 ft social distancing measure. These rooms ranged from small to medium-sized classrooms to large auditoriums and spaces not used as classrooms before, such as the ballroom and event spaces.

### Spatial data created for fixed-seating room

**Fig. 4:**
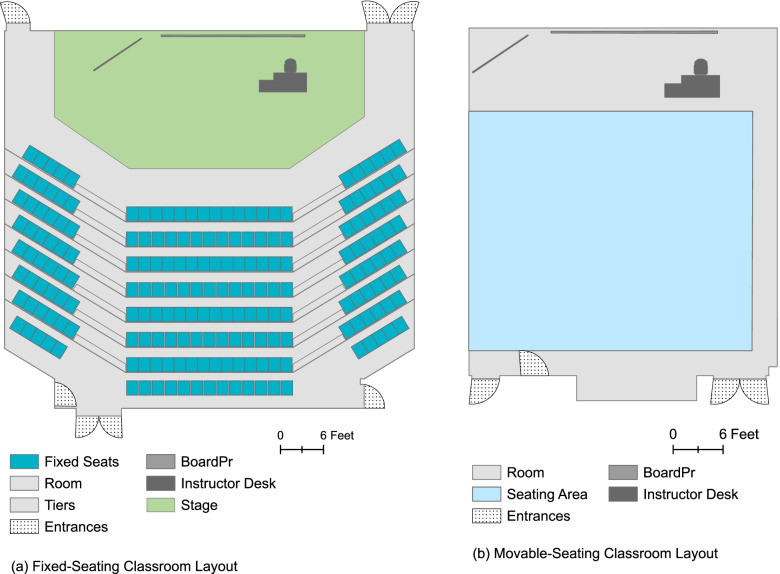
Spatial data created using DWG and JPEG files for classrooms

For illustration purposes, we selected a classroom that posed maximum challenges in digitization (Fig. 4(a)). The available DWG file provided basic floor plan data containing the room outline, tiers, entrances, and the stage for this particular room. The board and instructor desk were copied from other rooms, and included in the model to assist the map viewer in understanding the room’s layout. Since the current seating arrangement was unavailable in DWG files, JPEG photos were used as a reference to create the seating arrangement. The photos displayed the number of rows and the number of seats in each row. We used ArcGIS Fishnet tool to model this seating arrangement. It facilitated to replicate the arrangement in photos using dimensions of similar seats in other classrooms. Following the placement of seating, the attribute table was updated to give each seat a unique ID, define whether it was movable or fixed, and specify if it was handicap accessible.

### Spatial data created for movable-seating room

Like fixed-seating rooms, the room outline and entrances were digitized using DWG files. Additionally, a seating area was created to delineate the space within the room where movable seating could be placed (Fig. 4(b)). The size and placement of the seating area was determined following the demarcation of – the teaching area near the board and projector screen, and the passages along the sides of the room for entering and exiting the room. The teaching area encompassed an area 10 ft from the board and went across the width of the classroom whereas the passages were 3 ft in width. The remaining area within the classroom represented the seating area. An advantage of having movable seating classrooms was that detailed floor plans information or photos were not required because the current seating arrangement within the room could be easily altered to accommodate seating placement recommendations.

### Social distance planning for fixed-seating classrooms

We examined the proposed OBM with more than 20 classrooms that were larger than 1200 square feet (sq. ft.). We gave due consideration to errors that could arise from the slight shifting of fixed seats because of the projected coordinate system or manual errors in the digitization process. Therefore, the revised capacities with a social distance of 6 ft, 5.8 ft, 5.5 ft were applied in the OBM and compared with the original capacity. It is important to note that the original arrangement of seats was decided beforehand in the fixed-seating room. Therefore, the size of the seat, the range between seats and the seating arrangement would impact the seating capacity differently.

The results for four different sizes of classrooms are provided in Table 1. The original capacity ranged from 68 to 250 students among these classrooms; in general, a larger classroom indicated more seating capacity except for room S100, which covered an area of 3547.23 sq. ft. with 197 seats accommodation. An advantage in this case was the space available between the seats was more, and therefore it allowed a more flexible adjustment when social distancing was required. Room S100 also showed the highest utilization rate of 25.8%. Visualization of results showed that when the adjusted seating capacity between different social distances was the same, the seat arrangement was entirely the same or had only minor differences, and there was a significant difference in seating arrangement when the capacity varied.

**Table 1:** Results for fixed-seating classrooms with different social distances

Room	Area (Sq. Ft.)	OC	Revised Capacity (RC) for			UR^a^ (%)
			6.0 ft.	5.8 ft.	5.5 ft.	
M154	1279.53	68	15	15	17	22.06
S111	1525.72	134	27	29	30	20.14
SC208	2950.08	250	39	40	52	15.60
S100	3547.23	197	51	51	52	25.80

^a^Utilization rate (UR) is the percentage of revised capacity for 6 ft social distance to the original capacity (OC)

### Social distance planning for movable-seating classrooms

In this study, 68 classrooms with movable seats were modeled using the flexible model seating capacity tool. These rooms ranged from 600 to 7000 sq. ft. Out of total rooms, 70% had an area less than 2000 sq. ft. Thus, it could be implied that the flexible model was effective in planning seating capacity for moderate size classrooms for our case study. There were two possible ways to introduce social distance measurement in the flexible model – 1) person to person social distance; in this case, the center of the fishnet cell would represent the person as the origin of measurement. 2) object to object social distance; in this case, the distance was measured from the boundary of the fishnet cell. We considered both measurement styles in our study. The revised seating capacity with 6 ft from the center as well as the boundary of cells were obtained, and compared with the original seating capacity (Table 2). It was observed that the seating capacity of two rooms with the same area could be different. The possible reasons for this difference were – additional facilities or fixtures such as lab tools, building columns present in classrooms, reserved passages within the classroom for entrance or exit, limited availability of furniture, and different sizes of seats.

**Table 2:** Results for movable seating classroom obtained using different measurement techniques for social distancing

Room Number	Area (Sq. Ft.)	OC	RC^a^	RC^b^	UR^a^(%)	UR^b^(%)
SH126	715.700	20	12	6	60.00	30.00
W201	2281.939	108	24	20	22.22	18.52
M342	1391.884	NA	30	20	NA	NA

RC - Revised capacity

UR - Utilization rate is the percentage of revised capacity for 6 ft social distance to the original capacity (OC)

NA - Information not available

^a^ 6.0 ft. distance is measured from center of the fishnet cell

^b^ 6.0 ft. distance is measured from boundary of the fishnet cell

A set of results for three classrooms with areas 715.7 sq. ft., 2281.939 sq. ft., and 1391.884 sq. ft. is provided in Table 2. Each classroom presented a different scenario for study. The room SH126 was relatively smaller than others and had an original seating capacity of 20 students. However, it had the highest utilization rate (UR^1^) of 60% with a revised seating capacity equal to 12 students. This was because unlike fixed seats, movable seats could be rearranged in the available seating area to accommodate maximum seating capacity with given constraints. In our fixed seating model, the constraint was to maintain a social distance of 6 ft between the centers of each seat, instead of using the two different distance measures introduced in the flexible model.

**Fig. 5:**
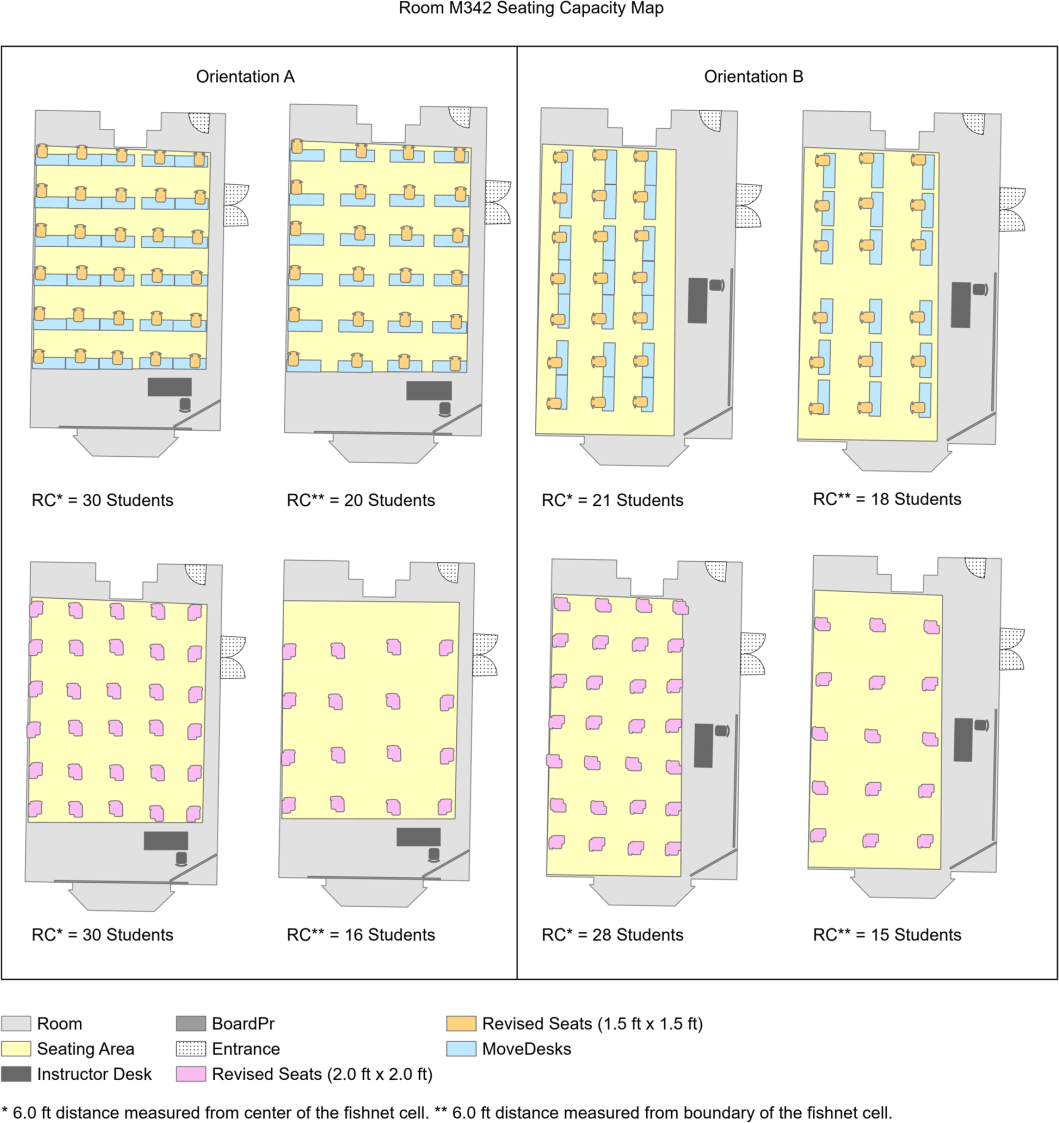
Revised seating capacity map for room M342

For a few rooms, such as M342, the original seating capacity was not available (NA). In this case, the utilization rates were not calculated. This exception was valid for spaces such as the ballrooms, library, and event spaces proposed to be modeled as classrooms during the pandemic. As the original furniture plan was not available for these rooms, we had more flexibility in designing the seating arrangement. We modeled two seating arrangements using a fishnet cell size of 1.5 ft. × 1.5 ft. and 2.0 ft. × 2.0 ft. The dimensions of the majority of available seats were within this range. We also studied orientation A and B with the instructor desk and teaching board aligned to shorter wall span and longer wall span, respectively (Fig. 5). It was observed that the position of the instructor desk and board significantly impacted the seating capacity due to reserved space. Additionally, the types of furniture such as stool or tablet arm chairs, also impacted the seating capacity.

Figure 5 shows that seats with 2.0 ft. × 2.0 ft. dimensions which were movable tablet arm chairs, provided a seating capacity of 28 students. Whereas seats with 1.5 ft. × 1.5 ft. dimensions which were stool chairs that required a separate desk for writing purpose, decreased the seating capacity to 21 students because of insufficient classroom space for the desk. These results showed that the flexible model seating capacity tool could be used effectively to consider various possible seating arrangements.

## Discussion and conclusion

Historically, GIS in building management has been used for the generic construction stages of building, operation and management, and demolition (Song et al., 2017). However, it has evolved from the conventional sense of building management, to better equip the human equation of safety. Using GIS in architecture, engineering, and construction provides spatial statistical methods of modeling data and problems, which is crucial to understanding the vast complexities and scenarios which go into creating a building. In this paper, we presented a custom GIS-based technique to address the social distancing measure in the classroom during the COVID-19 pandemic. Our case study demonstrated a classroom management technique for safe in-person learning. Both fixed model backtracking and flexible model seating capacity tools efficiently calculated revised seating capacity for classrooms by incorporating the user-defined social distance measure. These tools were custom developed to run within ArcGIS environment. The presented workflow and the supplementary pseudo codes of these tools can be readily utilized to create stand-alone executables using Python script. The outcome of this research has been disseminated internally to the University administration and the University’s public health workgroup. The seating capacity maps have been very helpful in guiding the reopening planning process in Fall 2020.

Though the COVID-19 pandemic will end the disease might continue to exist. Research studies in public health and medicines also suggest high possibilities of pandemics or epidemics in the future. Thus, it is crucial to have a contingency plan. As maintaining social (physical) distancing together with other measures such as using face masks and maintaining hygiene proved to be effective in curtailing the spread of such diseases, our GIS-based technique could be considered as a contingency plan for revised seating capacity during a pandemic, epidemic, or other public health emergencies. We should note that the policy of social distancing in different phases during a pandemic may change. Our classroom seating tool has been designed to be adaptable in a post-pandemic classroom design by allowing users to choose the different distance parameters. Recently, a large ceremony event was successfully hosted in one auditorium and 3 ft social distance was chosen to allow more people to attend the event while safely distanced. One future direction for post-pandemic classroom seating planning is to develop a web classroom management system that could support better classroom seating planning and user interaction allowing students to take a seat reservation, and query from the Internet or through a mobile device. We also propose to extend the applicability of our GIS-based classroom management system to other spaces. For instance, our fixed model backtracking tool could be used to calculate seating capacity for indoor gathering places such as movie theaters, restaurants, and stadiums. Outdoor spaces might not require face masks mandates while maintaining a social distance could be beneficial. Our flexible model could also be used to design seating arrangements and capacities for outdoor gatherings. Another future research direction is to demonstrate the extended applicability of a GIS-based classroom management system in other seating planning settings.

## Supplementary Information

**Additional file 1: Appendix A** – Supplementary Information.

## Data Availability

The datasets generated and analyzed during the current study are available in the Classroom repository on GitHub. https://github.com/truptilk/Classroom.
