# Supplementary material for: GIS-based classroom management system to support COVID-19 social distance planning
Source: Comput Urban Sci. 2022 May 28;2(1):11. doi: 10.1007/s43762-022-00040-3 (PMC9143716; doi:10.1007/s43762-022-00040-3)
Supplement: Supplementary file 1 — Additional file 1: Appendix A – Supplementary Information. [file 43762_2022_40_MOESM1_ESM.docx]

**Appendix A – Supplementary Information**

**1. Description of Data**

The classroom layouts were created using multiple shapefiles in ArcGIS software. Table A1 provides detailed information on attribute tables for shapefiles. The distinguishing field in this data was *SeatingType* that determined the algorithm utilized for calculating revised seating capacity.

Table A1. Description of shapefiles created to represent classrooms.

| Shapefile  Name | Shapefile Description | Field Name | Data Type | Field Description |
| --- | --- | --- | --- | --- |
| Room | Delineates room outline and area | RoomID | String | Room number |
| Entrance | Delineates entrances to the room | EntranceID | String | Entrance IDs (A, B, C, etc.) for possible routing. |
|  |  | Accessible | Short integer | Determine whether a doorway can be used as an entrance/exit (1 = yes; 0 = no). |
| AllSeats | Represents fixed and movable seating | AllSeatID | Short integer | Unique seat ID to run algorithm. |
|  |  | SeatingType | String | Type of seat (fixed or movable). |
|  |  | HandicapAc | Short integer | Define whether seat is handicap accessible. |
| SeatingArea | Delineates area for movable seat placement | Reference | String | Seating area polygon |
| FixedDesk | Represents fixed desks | FixDeskID | Short Integer | Fixed desk unique ID |
| MoveDesk | Represents movable desks | MoveDeskID | Short integer | Movable desk unique ID |
| BoardPr | Represents marker boards, projector screens | Reference | String | Define whether an object is a board or projector screen |
| Teaching | Represents instructor desk and chair | TeachRef | String | Define the instructor chair and instructor desk |
| LabTools | Delineates areas used for lab work | LabRef | String | Describe tools and features within labs |
| Tier | Represents changes in elevation within the room | TierRef | String | Tiers (Tier 1, Tier 2, Tier 3, etc.) if the room has changes in elevation. |
|  |  | RoomID | String | Room number |
| Other | Represents unknown objects in the floor plan | Reference | String | Description of other objects within the room. |

**2. Pseudo code for Optimized Backtracking Model**

In this paper, we applied an optimized backtracking model (OBM) to solve the maximum seating capacity problem for the fixed classroom. From the perspective of searching, the backtracking algorithm enumerates a set of partial candidates step by step, where all the potential solutions are tested, and a valid solution forwarded as a selection. During the process, if the selection is found to be unsatisfied, it will be abandoned and then, the process will track back to its previous step or even more steps and test the next available solution. Consequently, one of the two results will occur after operating the previous steps repeatedly: 1) One or more existing solutions to the whole set is found; 2) the question is unsolvable even though all possible steps have been tried (Fig.A1).

Generally, the eight queens puzzle is used to be an illustration for the backtracking algorithm. The eight queens puzzle requires the arrangements of eight chess queens on a standard chessboard of eight rows and columns, in which no two queens will share the same row, column, or diagonal. Ideally, we can see this puzzle as a geographic problem as: in a size of 8*8 space, how the eight queens can be arranged under the constraint rule that the same row, column or diagonal for two queens is forbidden. Moreover, the result can be taken as a solution for the arrangement problem. Meanwhile, the maximum capacity for this size of 8*8 space has already been implied during the process and it is noticeable that the maximum capacity would be indicated even if an unavailable capacity was required (e.g., require to put 9 queens in an 8*8 chessboard and the arrangement of 8 queens will be the output). Therefore, we applied this principle to the proposed fixed model backtracking tool.


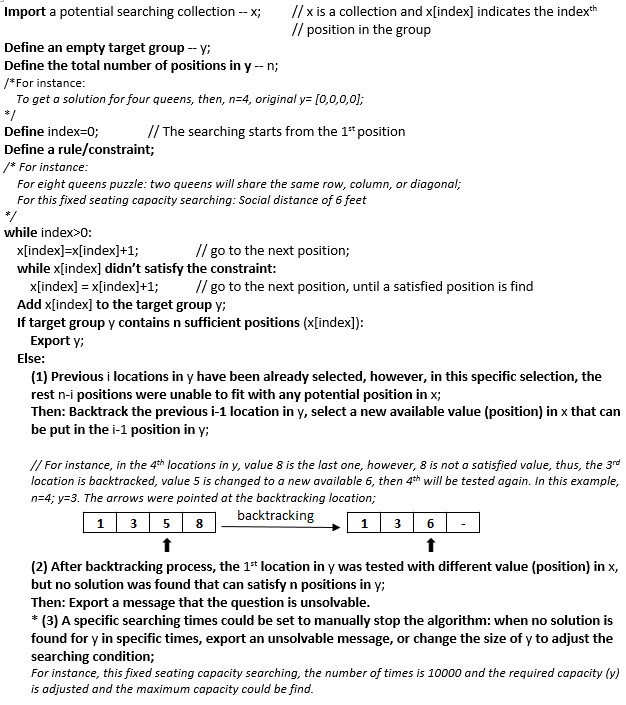


Fig.A1 Pseudo code for backtracking algorithm and optimized backtracking model (OBM)

**3. Flexible Model Seating Capacity Tool**

The flexible model seating capacity tool is easy to use and requires minimal data inputs from users. The two prominent user specified inputs required by the tool are – polygon shapefile representing seating area within a classroom, and distance required to be maintained between each seat. A crucial practice in slowing the spread of COVID-19 is maintaining physical distance from others. The tool ensures that each seat in the revised classroom furniture plan is at specified safe distance. Geoprocessing a huge dataset manually is time consuming, and it may incur errors in results. These limitations were overcome by the seating capacity tool. The tool is mainly based on three pre-defined ArcPy functions. These functions are – CreateFishnet_mangement() , Buffer_analysis(), Clip_analysis(). The algorithm was realized by calling the ArcPy site package in Python script. The pseudo code for seating capacity tool is described in Fig.A2.

The first process performed by the tool is creating a fishnet grid for a user specified polygon shapefile. The tool prompts the user to enter cell size - width and height of each cell. The primary output of CreateFishnet_mangement() is a rectangular cell grid. The algorithm is designed to evaluate each cell in the grid for its suitability with respect to the COVID-19 social distancing measure. The secondary output is sampling points created at the center of each cell. These sampling points called labels are used by the algorithm to define suitable and unsuitable cells for seating.

The next sequential processes performed by the tool are - buffering the fishnet cell and clipping sampling points. These tasks are carried out by using Buffer_analysis() and Clip_analysis() functions. The tool provides flexibility in using different values and units of measurements for social distances to perform buffer analysis. Sort_management() helps in identifying the first suitable cell in the fishnet.


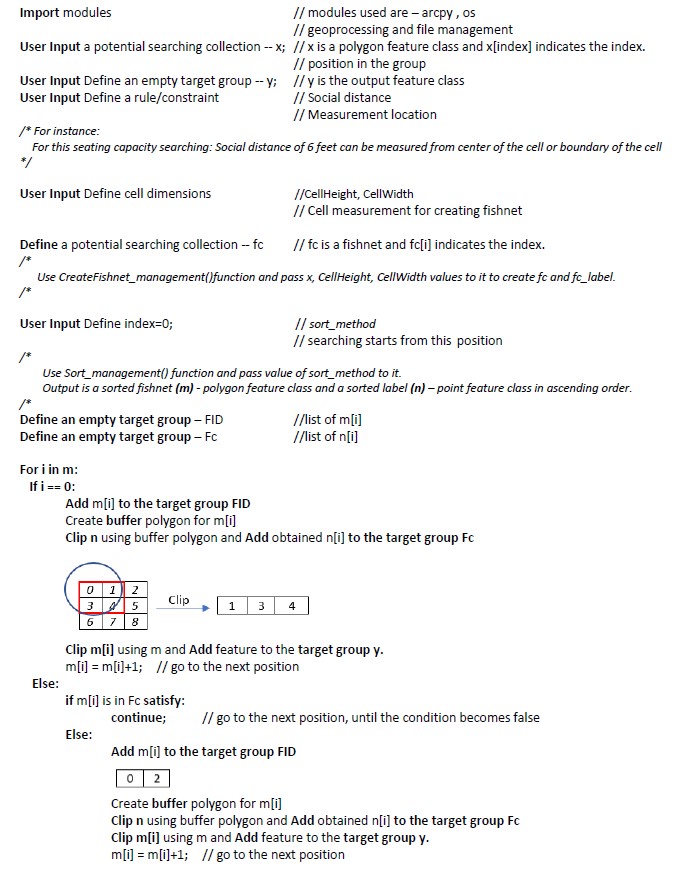


Fig.A2 Pseudo Code for Seating Capacity Tool Algorithm

Buffer polygon is created around this first cell and sampling points within the buffer polygon are clipped. The cells containing these sampling points are reserved as unsuitable locations for seating. The next subsequent fishnet cell outside the buffer polygon is selected by the algorithm to perform buffer analysis, running the algorithm until the last cell of the fishnet is evaluated. The cells over which buffer analysis is performed get reserved as suitable or safe locations for seating. The output feature class obtained from the seating capacity tool is a polygon shapefile representing revised furniture plan for the classroom.

3.1. Tool Parameters

The user interface for the seating capacity tool is shown in Fig.A3. It uses seven parameters to model classroom seating capacity. The primary function of each parameter is described in Table A2. The tool allows the user to perform analysis on any polygon shapefile.

**
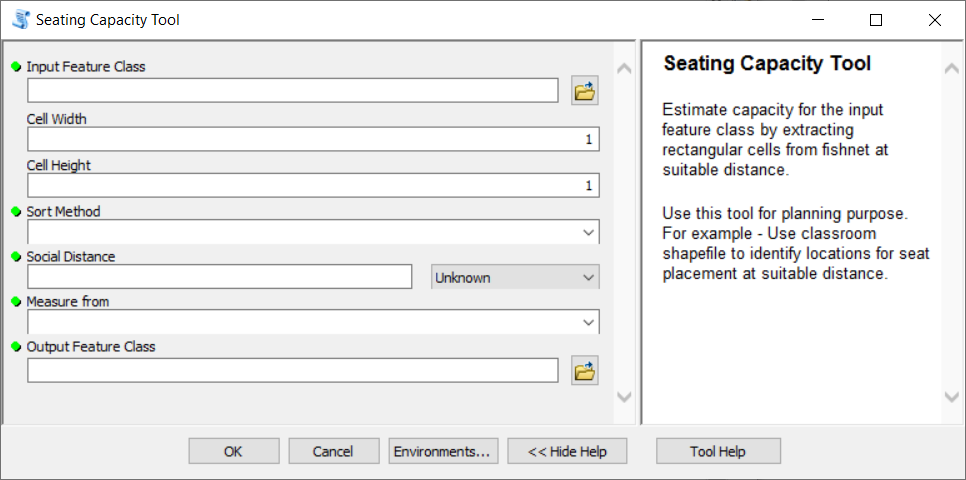
**

Fig.A3 Seating Capacity Tool User Interface in ArcGIS software

Table A2. Description of Seating Capacity Tool Parameters

| Parameter | Explanation | Data Type |
| --- | --- | --- |
| Input Feature Class | Specifies the extent of the fishnet. | Polygon Feature Class |
| Cell Height | Determines the height of each cell of the fishnet. | Double |
| Cell Width | Determines the width of each cell of the fishnet. | Double |
| Sort Method | Determines the sequence for creating buffer polygons around cells.   - UL – From the upper left corner of the fishnet. - UR – From the upper right corner of the fishnet. - LL – From the lower left corner of the fishnet. - LR – From the lower right corner of the fishnet. | String |
| Social Distance | Specifies the distance around the cell that will be buffered. | Linear Unit |
| Measure From | Specifies the starting point to apply social distance.   - CENTER – The social distance is measured from the center of the cell that will be buffered. - BOUNDARY – The social distance is measured from the boundary of the cell that will be buffered. | String |
| Output Feature Class | Specifies the name and workspace for output feature class. | Shapefile |
